# Coronary Computed Angiography and Coronary Artery Calcium Score for Preoperative Cardiovascular Risk Stratification in Patients Undergoing Noncardiac Surgery

**DOI:** 10.3390/jcdd12040159

**Published:** 2025-04-17

**Authors:** Ioannis Kyriakoulis, Sriram S. Kumar, Georgios D. Lianos, Dimitrios Schizas, Damianos G. Kokkinidis

**Affiliations:** 1 Faculty of Medicine, School of Health Sciences, University of Thessaly, 41100 Larissa, Greece; kyioannis@uth.gr; 2 Department of Medicine, Jacobi Medical Center, 1400 Pelham Parkway South, 3N1, Suite B, Bronx, NY 10461, USA; sunilkus@nychhc.org; 3 Department of Surgery, University Hospital of Ioannina, 45110 Ioannina, Greece; glianos@uoi.gr; 4 Department of Surgery, National and Kapodistrian University of Athens, Laikon General Hospital, 11527 Athens, Greece; dschizas@med.uoa.gr; 5 Heart and Vascular Institute, Yale New Haven Health, Lawrence and Memorial Hospital, New London, CT 06320, USA

**Keywords:** coronary artery calcium score (CACS), coronary computed tomography angiography (CCTA), major adverse cardiovascular events (MACE), noncardiac surgery, preoperative risk stratification

## Abstract

Perioperative and long-term postoperative major adverse cardiovascular events (MACE) are a leading cause of morbidity and mortality in patients undergoing noncardiac surgery. In selected high-risk patients, when information about cardiovascular status may influence surgical decisions, preoperative risk stratification is reasonable, with stress imaging being the preferred method. Coronary computed angiography (CCTA) and coronary artery calcium score (CACS) offer direct anatomical assessment of atherosclerotic coronary arteries and help gauge the extent and severity of coronary artery disease. Strong evidence supports that CCTA and CACS, either alone or in combination, are reliable methods for assessing the risk of both perioperative and long-term postoperative MACE, often demonstrating equal or superior prognostic performance compared to traditional imaging tools. Moreover, integrating CCTA or CACS into standard preoperative imaging protocols further enhances perioperative risk prediction and improves the ability to accurately stratify patients. Future research is needed to better define the role of CCTA and CACS in preoperative cardiovascular risk evaluation of patients undergoing noncardiac surgery.

## 1. Introduction

Cardiovascular perioperative complications are common and are associated with increased 1-year postoperative mortality [1]. The incidence of postoperative major adverse cardiac events (MACE) varies widely between different examined cohorts, ranging from 1.8% to 25% [2,3,4]. Older patients with existing cardiovascular disease or comorbidities (diabetes mellitus, kidney dysfunction, etc.) are at higher risk for perioperative cardiovascular complications and cardiac death [5,6,7]. Importantly, poor functional status and reduced exercise capacity—defined as metabolic equivalents (MET) of less than 4—have been consistently linked with increased risk of poor postoperative outcomes [8]. This high-risk population should undergo preoperative risk evaluation to determine whether further strategies (guideline-directed medical therapy, revascularization, etc.) should be employed to reduce the risk and if the benefits of the surgery override the relevant risks. The ACC/AHA guidelines state that stress testing can help stratify risk and guide management in patients suspected of high-risk ischemia [8]. In a similar manner, the 2022 European Society of Cardiology (ESC) supports stress imaging in high-risk noncardiac surgery with poor functional capacity, high likelihood of coronary artery disease (CAD), high clinical risk according to the Revised Cardiac Risk Index (RCRI), or previous coronary revascularization [9].

Coronary computed tomography angiography (CCTA)—an important diagnostic tool for noninvasive anatomic evaluation of CAD—has been extensively investigated as an imaging modality for cardiovascular risk stratification. Both the Appropriate Use Criteria for Cardiac Computed Tomography (2010) [10] and the 2014 ACC/AHA guidelines [11] stated that there are no appropriate indications for using CCTA in preoperative evaluations for noncardiac surgery. However, in light of new evidence, the latest 2022 ESC guidelines and most recent 2024 ACC/AHA guidelines recognize the role of CCTA in assessing suspected CAD in select patients [8,9]. Indeed, numerous observational studies have demonstrated that CCTA as well as coronary artery calcium score (CACS) measurement are reliable options for perioperative risk stratification, yielding outcomes comparable to those of traditional imaging methods [12,13,14,15]. In this narrative review, we explore whether CCTA or CACS can effectively stratify the risk of patients prior to noncardiac surgery.

## 2. Perioperative Risk Stratification

### 2.1. CCTA

CCTA is recommended as the initial test for evaluation of patients with low-to-intermediate risk for CAD [16]. Thanks to its high negative predictive value (NPV), CCTA acts as a gatekeeper for invasive coronary angiography (ICA) [17].

In the surgical population, given that no existing evidence has demonstrated that preoperative revascularization reduces postoperative MACE and cardiac mortality [18], the 2024 ACC/AHA guidelines recommend against routine CCTA preoperative imaging and support the use of CCTA in select high-risk patients [8]. CCTA has shown comparable or even superior performance compared to tools like the RCRI and other imaging modalities, such as dobutamine stress echocardiography (DSE) or myocardial perfusion imaging (MPI), for accurately estimating cardiovascular risk and predicting postoperative MACE [19,20].

Previous studies have demonstrated a higher risk of MACE in patients diagnosed with obstructive CAD (stenosis > 50% or >70% on CCTA) [19,21,22,23]. Ahn et al. investigated 239 patients undergoing intermediate risk noncardiac surgery and reported a more than five-fold increase in postoperative events, including cardiac death, acute coronary syndrome, pulmonary edema, and arrythmias, in patients with significant (>50%) coronary stenosis on CCTA [21]. Similarly, Hwang et al. reported an increased risk of perioperative major cardiac events in patients with significant stenosis. There was an exponential risk with higher stenosis severity (OR: 6.1, 95% CI: 2.7–14 for stenosis > 50% vs. OR: 8.4, 95% CI: 3.6–19.6 for stenosis > 70%) [22].

Significant stenosis on CCTA has also been found to have an additive value on the prognostic performance of RCRI, a commonly used risk model that uses six different clinical risk predictors of perioperative cardiovascular events [24]. Hwang et al. found that the combination of segment involvement score > 3, Duke jeopardy score > 0 and RCRI resulted in a significantly increased area under the receiver operating characteristic curve (AUC) compared to RCRI alone for diagnosing perioperative cardiac events (AUC: 0.757 vs. AUC: 0.631, *p* = 0.003) [22]. Furthermore, CCTA findings can significantly reclassify the risk of perioperative major cardiac events in patients categorized as having RCRI risk levels 2 and 3. For those with significant CCTA findings, the odds of experiencing such events can increase 3- to 17-fold compared to patients with non-significant findings (*p* < 0.05). Based on these findings, the authors highlighted the usefulness of CCTA in further stratifying the risk of patients with an RCRI of 2 or 3 [22].

Li et al. investigated the impact of CCTA findings on surgery cancellation in a study involving 841 older patients (mean age 69.5 ± 5.8 years) with unknown or suspected CAD. Their findings revealed that the likelihood of canceling scheduled surgeries increased with the severity of coronary stenosis and the number of affected coronary arteries [23].

On the other hand, while CCTA possesses excellent ability to exclude nonsignificant CAD, false-positive findings due to calcium blooming artifacts and coronary lumen caliber underestimation are common [25]. Sheth et al. investigated the composite outcome of perioperative cardiovascular death and non-fatal myocardial infarction (MI) in patients with or at risk of atherosclerotic CAD undergoing noncardiac surgery [19]. The study found that combining CCTA findings with the RCRI, compared with RCRI alone, correctly reclassified as being at higher risk 17 out of 77 patients who experienced a perioperative event (*p* < 0.001). However, using CCTA also led to an overestimation of the risk of 98 out of 923 patients who did not experience a perioperative event. The last finding is of great importance since overestimation of the cardiovascular risk in patients scheduled for surgery imposes great risks, including redundant further cardiovascular evaluation and potentially detrimental surgery delay or even cancellation. Studies that have evaluated diagnostic and prognostic significance of preoperative CCTA are described in Table 1.

### 2.2. Coronary Artery Calcium Score

CACS is a measure of atherosclerosis burden in the coronary arteries and acts as an indicator of CAD. Closely correlated with the severity of CAD, elevated CACS serves as an independent predictor for mortality and MACE in both symptomatic and asymptomatic patients [34,35].

CACS—alone or in combination with CCTA findings—has been also investigated as a method of preoperative risk stratification. The existing literature attests that an elevated CACS is associated with an increased risk of perioperative cardiovascular events. Studies that have evaluated diagnostic and prognostic significance of preoperative CACS are described in Table 2.

In 2001, Mahla et al. examined the preoperative CACS, measured by electron beam computed tomography, in 51 patients undergoing elective vascular surgery. Six patients with elevated postoperative cardiac troponin T levels had a median CACS 2.5 times higher than the remaining 45 troponin-negative patients (2080 vs. 810; *p* = 0.021), suggesting a possible association between preoperative CACS and postoperative cardiac cell injury [42].

Choi et al. investigated 2554 patients having a non-gated chest CT performed within one year before surgery [37]. The authors employed an estimated coronary calcium burden (ECCB) score ranging from 0 to 9 with ECCB: 3–9 scores indicating severe disease (≥50% of the total artery length calcified) in one or two vessels. The authors found that higher coronary calcium burden was associated with a stepwise increase in the rate of postoperative major clinical events (MCE), defined as perioperative mortality and MI (ECCB 0: 2.9%, ECCB 1–2: 3.7%, ECCB 3–5: 8.0%; ECCB 6–9: 12.6%, *p* < 0.001). The odds for perioperative clinical events were twice as high in patients with ECCB > 3 compared to patients with ECCB < 3 (adjusted OR: 2.11, 95% CI: 1.42–3.12). Finally, the study demonstrated that implementing ECCB score in an RCRI model resulted in a significant improvement of the AUC for prediction of MCE (from 0.675 to 0.712, *p* = 0.018), with a net reclassification improvement of 0.428 (95% CI, 0.254–0.601, *p* < 0.0001). Of note, all-cause death rather than cardiac death was included in the composite primary outcome.

In another study of 4491 patients with lung cancer who underwent intermediate-risk surgery, the presence of coronary calcification (CACS ≥ 1) was associated with increased risk for perioperative cardiovascular events (OR: 1.75, 95% CI: 1.14–2.68) after adjustment for relevant variables [38]. Finally, in a meta-analysis of eleven studies including 3480 patients undergoing noncardiac surgery, a ten-fold increase in risk for perioperative MACE was seen in patients having severe coronary calcifications (CACS ≥ 1000 vs. CACS < 1000, OR:10.4, 95% CI 1.6–69.7) [43].

In terms of prognostic performance, a previously used CACS cut-off of 113 demonstrated an NPV of 97% for postoperative cardiac events in patients undergoing intermediate-risk noncardiac surgery, highlighting the strong ability of CACS to identify patients at low risk for perioperative events [21].

It remains unclear whether preoperative CACS measurement has different predictive value in men versus women. This is an important gap, as prior large cohorts have shown that women with CACS > 100 have significantly higher cardiovascular mortality compared to men [44]. No available studies in the existing literature have directly compared CAC burden and postoperative outcomes stratified by gender, but most available analyses were adjusted for sex using multivariable Cox models, which may partially account for these differences. Notably, women are more likely to present with ischemic equivalents (e.g., fatigue, dyspnea, etc.), making symptom-based evaluation challenging [45]. In such cases, CACS and CCTA may provide more reliable risk assessment and improve diagnostic accuracy.

There are insufficient data in the literature to determine whether CACS, CCTA, or a combination of both is superior for risk stratification of perioperative cardiovascular events. While CACS possesses several advantages over CCTA (easier to perform, no risk for contrast induced allergy/nephropathy, etc.), it cannot adequately evaluate the coronary anatomy and the presence and the extent of non-calcified stenoses. Future comparative studies are needed to address this question.

**Figure 1:**
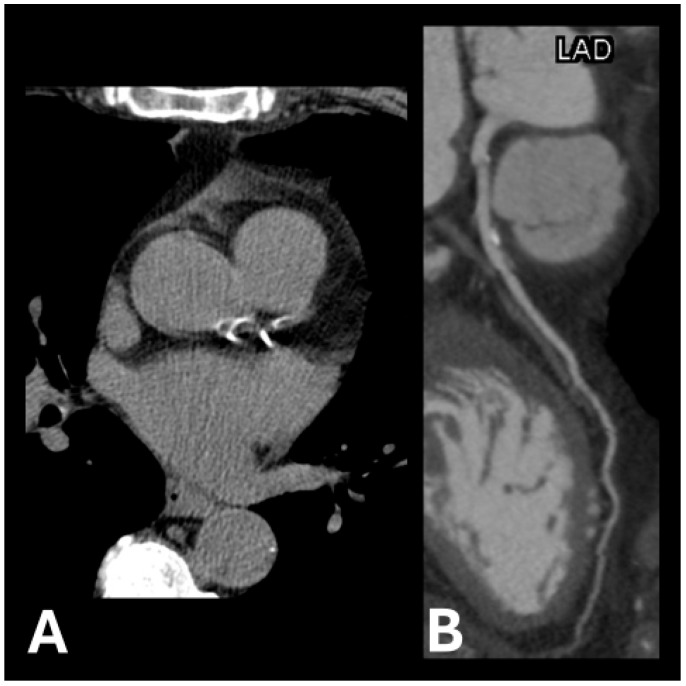
Examples of calcification plaque(s) in (**A**) left anterior descending artery and left circumflex artery and (**B**) left anterior descending artery.

**Figure 2:**
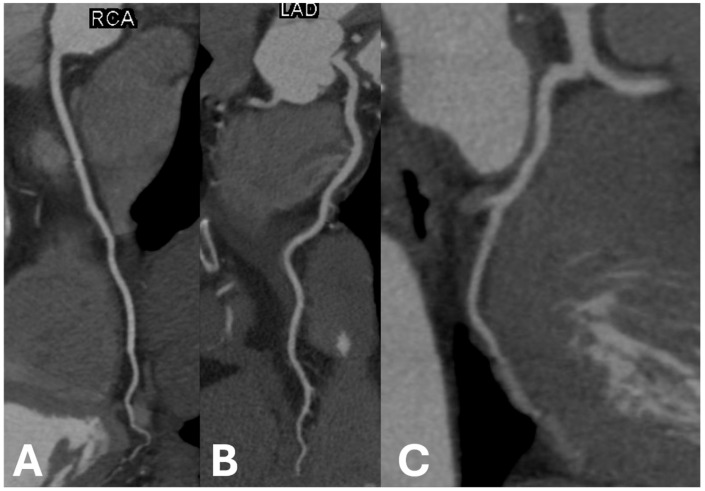
Examples of non-calcified coronary arteries in (**A**) right coronary artery, (**B**) left anterior descending artery, and (**C**) left circumflex artery.

Example images of calcified and non-calcified coronary arteries are shown in Figure 1 and Figure 2, respectively.

### 2.3. Fractional Flow Reserve–Computed Tomography (FFR-CT)

FFR-CT is a noninvasive diagnostic tool with great correlation and agreement with invasive FFR and better performance than CCTA alone in diagnosing CAD [46]. Ma et al. investigated the predictive value of machine-learning based FFR-CT in patients scheduled for lung cancer surgery [47]. FFR-CT ≤ 0.8 (compared to FFR-CT > 0.8) was associated with 10-fold increased odds for MACE in the perioperative period. Furthermore, when assessing the accuracy for prediction of MACE, FFR-CT outperformed CCTA findings (AUC: 0.737 vs. AUC: 0.524). Given its higher NPV for CAD, FFR-CT could potentially act as a gatekeeper for preoperative ICA when DSE or CCTA results are suspected to be false-positive.

## 3. Long-Term Outcomes

CCTA findings and CACS have been studied for risk stratification and prediction of long-term cardiovascular events, with coronary atherosclerosis burden emerging as a negative predictor. With a median follow-up of 4 years, Sampaio Rodrigues et al. reported a hazard ratio of 5.8 (95% CI 1.6–20.6) for MACE when significant CAD (stenosis > 50%) was identified preoperatively on CCTA [14]. Another study found a 12-fold increase (OR: 11.97, 95% CI: 5.79–24.75) in MACE for patients with severe coronary calcification (CACS > 400) or obstructive CAD in a follow-up period of 5 years [13]. Finally, when assessing the diagnostic value of CCTA, Cassagneau et al. found a 95% and 100% NPV of CCTA for MACE and coronary events (increase in creatine kinase or troponin, MI, postoperative coronary artery revascularization, etc.), respectively [30]. No significant differences were observed between the diagnostic performances of DSE and CCTA.

## 4. Revascularization Prior to Noncardiac Surgery

The usefulness of CCTA and CACS findings should be carefully considered given the lack of evidence supporting a strategy of routine revascularization before noncardiac surgery in stable CAD patients [48]. Two large trials (CARP and DECREASE-V) showed no benefit of prophylactic revascularization for short-term outcomes (death, MI, etc.) or long-term survival in vascular surgery patients [18,49]. Of note, left main CAD patients were excluded from both trials. A sub-analysis of the CARP trial, however, showed that revascularization in patients with anterior ischemia on stress imaging reduced long-term death and MI risk [50]. The 2024 ACC/AHA guidelines advise against routine revascularization before noncardiac surgery and recommend it only for traditional indications (e.g., unstable angina, left main disease, or high-risk anatomy) [8]. Perhaps, better stratification of preoperative cardiovascular risk, implementing clinical variables and imaging results, could help identify which patients might benefit from revascularization versus those who might be harmed by unnecessary procedures or delayed surgery.

## 5. Specific Types of Surgeries

Besides research on patients undergoing noncardiac surgery in general, there are also studies examining specific operation types. One population that has been particularly well studied is patients preparing for liver transplantation (LT) surgery. Patients with cirrhosis (especially metabolic-associated fatty liver disease-associated) are considered as high risk for CAD due to both traditional (obesity, metabolic syndrome, diabetes, and hypertension) and nontraditional risk factors (related to end-stage liver disease) [51]. LT recipients face a higher risk of cardiovascular death and ischemic events compared to the general population matched for age and sex [52]. For this reason and to select the most suitable candidates, LT patients undergo routine cardiac preoperative evaluation, commonly with stress electrocardiography, stress echocardiography, or MPI studies [53].

CCTA has been studied as an alternative imaging modality for preoperative evaluation of LT patients, with adequate ability of ruling out obstructive coronary stenosis [31,32]. More importantly, CACS has been shown to have great predictive ability of perioperative cardiovascular events. Kong et al. reported that patients with severe calcification (CACS > 400) had significantly increased odds for cardiovascular complications one month after LT (OR: 4.62, 95% CI: 1.14–18.72) [40]. Another study with 66 LT recipients undergoing both CACS measurement and ICA showed that a CACS of less than 400 (less than severe calcification) appeared to have a 100% NPV for obstructive CAD [36]. Consequently, the authors suggested there is no need for further CAD evaluation in patients with CACS < 400. A cutoff of 400 was also used by another study which reported a 100% NPV and estimated that 24% of their cohort could have avoided catheterization without missing any obstructive coronary disease [39].

Regarding vascular surgeries, patients undergoing intermediate-risk surgeries like carotid endarterectomy or endovascular aneurysm repair and high-risk surgeries like open aortic surgeries are usually risk-stratified based on traditional testing with echocardiography and MPI [29]. Prior studies evaluating the role of ICA and selective revascularization in patients undergoing vascular surgeries did not show significant benefit in outcomes [18,54]. However, compared to LT, fewer studies on the use of CACS and CCTA have been performed in this population. In a recent retrospective study of patients undergoing open peripheral artery bypass surgery, patients with known CAD (history of myocardial infarction or revascularization) and those with subclinical atherosclerosis determined by a positive CAC had similar postoperative troponin elevation and heart failure events [55]. Chang et al. evaluated the use of CCTA combined with stress perfusion CT in patients undergoing various vascular surgeries [29]. While CAD was common in these patients, there were only a few cardiovascular events overall, and perfusion defects did not predict perioperative cardiac events. Lastly, Vallier et al. showed that postoperative myocardial injury was better predicted by CCTA when compared to functional testing [56].

Studies on other specific surgery populations, such as bariatric surgery [27,33] and foot amputation in type 2 diabetics [12,28] have been also demonstrated sufficient CCTA and CACS performance in predicting postoperative outcomes.

## 6. CCTA and CACS in Relation to Other Imaging Modalities

### 6.1. Dobutamine Stress Echocardiography (DSE)

Data comparing CCTA with other imaging modalities for preoperative assessment were limited until the publication of the PANDA trial in 2020 [57]. In this prospective observational study, 215 patients with more than one clinical risk factor for perioperative cardiovascular events received both DSE and CCTA prior to noncardiac surgery. The authors employed different models for prediction of events, implementing RCRI scores, CCTA findings (≥50% stenosis and/or CACS ≥ 203), or DSE abnormal results. They found that combining CCTA findings with the RCRI score significantly enhanced risk stratification compared to the RCRI alone (*p* < 0.001), an improvement not observed with DSE. When DSE plus RCRI were compared head-to-head with significant stenosis on CCTA plus CACS > 203 plus RCRI, the latter model provided significantly better prognostic performance (AUC: 0.713 vs. AUC: 0.839, *p* = 0.027).

### 6.2. Single-Photon Emission Computed Tomography Myocardial Perfusion Imaging (SPECT MPI) and Stress Cardiovascular Magnetic Resonance Imaging (CMR)

Ghadri et al. assessed the added value of using CACS in combination with SPECT MPI. The study showed that using a cut-off of CACS ≥ 1314 improved the risk discrimination both when perfusion studies were normal and abnormal. Most importantly, CACS < 1314 combined with a normal perfusion study predicted lower risk for perioperative events than a normal perfusion study alone. However, this study did not directly compare the predictive value of CCTA with that of SPECT MPI [41]. Similarly, the VISION-CTA trial showed that the predictive accuracy of SPECT MPI for MACE improved significantly when CCTA results (stenosis ≥ 70%) were integrated into the risk evaluation [20]. Addition of pathological CCTA results to SPECT MPI led to an increase in NPV for MACE from 75% (CI 34.9–96.8) to 100% (CI 79.4–100). These findings highlight the potential additive value of incorporating CCTA and CACS into routine imaging methods.

CMR is another tool for evaluating myocardial ischemia and has demonstrated higher diagnostic sensitivity than SPECT in detecting angiographically significant CAD [58]. In a recent study of 669 patients undergoing MPI before intermediate- to high-risk noncardiac surgery, postoperative cardiac event rates were similar between patients who underwent stress CMR and those who had SPECT-MPI [59]. Due to the limited availability of supporting evidence, the 2024 ACC/AHA guidelines do not yet define a clear role for stress CMR in preoperative risk stratification [8]. Overall, there are insufficient data to conclude whether an anatomy-based approach (e.g., CCTA) or an ischemia-based approach (e.g., stress CMR or SPECT) provides superior prognostic value in the preoperative setting.

## 7. Conclusions

CCTA and CACS have consistently been shown to effectively predict the risk of both perioperative and long-term cardiovascular events. Future studies directly comparing CCTA with standard imaging methods are needed to clarify the optimal role of CCTA in preoperative cardiovascular evaluation for patients undergoing noncardiac surgery.

## Figures and Tables

**Table 1:** Studies evaluating the preoperative use of coronary computed tomography angiography.

Author	Year	Study Design	Total Patients, *n*	Age, Years Mean ± SD	Type of Surgery	Objective	Key Findings	Follow-Up Period
Sampaio Rodrigues et al. [14]	2024	R	229	66 ± 5	OLT (patients with intermediate to high pre-operative cardiac risk)	Assess the predictive value of CCTA for postoperative cardiovascular events	CAD-RADS ≥ 3 but not CACS significantly increased MACE risk	4 years
Shalaeva et al. [12]	2022	P	199	62 ± 7	Partial foot amputations (patients with type 2 diabetes and peripheralartery disease)	Assess the predictive value of CCTA for 1-year all-cause mortality	Two- and three-vessel obstructive CAD on CCTA increased all-cause mortality risk; any obstructive CAD was associated with higher MACE risk	1 year
Walpot et al. [26]	2022	P	735	70 ± 9	Noncardiac surgery (patients with atherosclerotic risk factors or a history of congestive heart failure)	Assess if subendocardial attenuation using coronary CCTA predicts perioperative MACE	Normalized subendocardial attenuation independently and incrementally predicted 30-day MACE in a model including RCRI and CAD severity	1 month
Kim et al. [13]	2021	R	628	53 ± 8	OLT	Assess the predictive value of CCTA for late postoperative cardiovascular events	Mixed plaque, obstructive CAD, 1- and ≥2-vessel CAD, and a CACS > 400 were significantly associated with MACEs within five years after liver transplant	5 years
Li et al. [23]	2020	R	841	70 ± 6	High-risk noncardiac surgery (patients with significant CAD)	Assess the predictive value of CCTA for predictors of significant CAD and the event of cancelling scheduled surgery	Surgery cancellations increased with stenosis severity and the number of obstructed major coronary arteries; postoperative medication use increased with stenosis severity	NA
Messerli et al. [27]	2017	P	54	49 ± 7	Bariatric surgery	Assess the predictive value of CCTA for postoperative cardiovascular events	Absence of coronary stenosis on CCTA ruled out long-term MACE with a 100% NPV	6.1 years (mean)
Fathala et al. [15]	2016	R	93	60 ± 9	Intermediate- and high-risk noncardiac surgery	Assess the predictive value of CCTA for postoperative cardiovascular events	Normal or nonobstructive CCTA excluded significant CAD and predicted favorable postoperative outcomes	Hospital discharge
Shalaeva et al. [28]	2016	P	179	60 ± 8	Trans-femoral amputation (patients with type 2 diabetes and peripheral artery disease)	Assess the predictive value of CCTA for perioperative MACE	Patients with ≥50% stenosis on CCTA had a higher 6-month MACE rate than those with normal or non-obstructive CCTA; patients with 3-vessel obstructive stenosis on CCTA had a higher 6-month MACE rate than those with two- or one-vessel obstructive CAD	6 months
Hwang et al. [22]	2015	R	844	67 ± 11	Noncardiac surgery	Assess the predictive value of CCTA for postoperative cardiovascular events	The addition of CCTA to clinical risk assessment improved perioperative risk stratification; absence of significant CCTA findings had high specificity and NPV for perioperative cardiovascular event risk, regardless of clinical risk	1 month
Sheth et al. [19]	2015	P	955	70 ± 9	Noncardiac surgery (patients with history or risk factors for CAD)	Assess the predictive value of CCTA for postoperative cardiovascular events	Compared to RCRI, CCTA improves risk estimation for perioperative cardiovascular death or myocardial infarction	1 month
Chang et al. [29]	2014	P	91	68 ± 9	Elective vascular surgery	Assess the predictive value of CCTA for postoperative cardiovascular events	No definitive conclusion was drawn on CCTA’s role in preoperative risk evaluation due to low event rates	1 month
Ahn et al. [21]	2013	R	239	70 ± 10	Intermediate-risk noncardiac surgeries	Assess the predictive value of CCTA for postoperative cardiovascular events	Significant coronary artery stenosis (>50%) and multivessel CAD were correlated with postoperative cardiac events; RCRI combined with CACS or multivessel disease predicted outcomes better than RCRI alone	1 month
Cassagneaua et al. [30]	2012	P	82	53 ± 10	OLT	Assess the predictive value of CCTA for postoperative cardiovascular events compared to DSE	Normal or nonobstructive CCTA had a 95% NPV for MACE and 100% for clinical coronary events; CCTA had a prognostic value comparable to DSE	1 year
Chae et al. [31]	2012	P	247	56 ± 7	OLT	Assess the clinical value of CCTA for postoperative cardiac events	3% of patients developed stress cardiomyopathy after surgery, but none experienced a cardiovascular event requiring emergency intervention	3 months
Jodocy et al. [32]	2012	R	54	56 ± 6	OLT	Assess the predictive value of CCTA for postoperative cardiovascular events	No cardiovascular events occurred during the follow-up period	9–15 months
Tognolini et al. [33]	2012	P	30	52 ± 15	Bariatric surgery	Assess the diagnostic value of coronary dual-source computed tomography (DSCT) in determining significant stenosis of coronary arteries	CCTA was more accurate than calcium scoring or traditional risk stratification in detecting severe CAD in this population	NA

Abbreviations: CACS, coronary artery calcium score; CAD, coronary artery disease; CAD-RADS, Coronary Artery Disease Reporting and Data System; CCTA, coronary computed tomography angiography; DSE, dobutamine stress echocardiography; DSCT, dual-source computed tomography; MACE, major adverse cardiovascular events; NPV, negative predictive value; OLT, orthotopic liver transplant; R, retrospective; RCRI, Revised Cardiac Risk Index; P, prospective.

**Table 2:** Studies evaluating the preoperative use of coronary artery calcium score.

Author	Year	Study Design	Total Patients, *n*	Age, Years Mean ± SD	Type of Surgery	Objective	Key Findings	Follow-Up Period
Groen et al. [36]	2024	R	149	58 ± 9	OLT	Assess the predictive value of non-gated CACS to identify low-risk patients for whom further cardiac imaging could be safely withheld compared to SPECT MPI, CCTA, or ICA and its correlation with perioperative mortality	The NPV of no or mild calcifications on CAC for obstructive CAD on CCTA, and ICA was 100%; additional cardiac imaging could have been safely avoided in ~75% of patients by reviewing existing CAC data	1 month
Choi et al. [37]	2023	R	2554	68 ± 13	Intermediate- to high-risk noncardiac surgery	Assess the predictive value of coronary calcium estimates from existing non-gated chest CT imaging for perioperative major clinical events	Higher estimated coronary calcium burden values were linked to stepwise increases in perioperative MACE, with additional risk classification improvement when added to an RCRI model	1 month
Shalaeva et al. [12]	2022	P	199	62 ± 7	Partial foot amputations	Assess the predictive value of CACS for 1-year all-cause mortality in type 2 diabetes patients with peripheral artery disease	Higher CACS was associated with an increased risk of all-cause mortality and MACE during follow-up	1 year
Yang et al. [38]	2022	R	4491	57 ± 11	Intermediate-risk lung cancer surgery	Assess the predictive value of prior non-gated CACS for perioperative cardiovascular events	CACS ≥ 1, and the number of calcified vessels were independently associated with perioperative cardiovascular events	Hospital discharge
West et al. [39]	2019	R	54	64 ± 6	OLT	Assess the predictive value of prior non gated CACS for clinically significant CAD compared to ICA	An Agatston score < 4 or Weston score < 2 excluded obstructive CAD, theoretically allowing 24% and 28% of patients, respectively, to avoid catheterization without missing significant cases	NA
Shalaeva et al. [28]	2016	P	179	60 ± 8	Trans-femoral amputation	Assess the predictive value of CACS for perioperative MACE in type 2 diabetes patients with peripheral artery disease	The postoperative event rate increased from 10% in patients with CACS 1–99 to 84% in those with CACS > 1000; patients with CACS = 0 had no MACE during the follow-up period	6 months
Kong et al. [40]	2015	R	443	52 ± 8	OLT	Assess the predictive value of CACS for early postoperative cardiovascular complications	CACS > 400 predicted early postoperative cardiovascular complications in OLT recipients	1 month
Ghadri et al. [41]	2012	P	326	71 ± 9	Elective noncardiac surgery	Assess the predictive value of CACS alone and in combination with SPECT MPI for postoperative cardiovascular events in patients with suspected increased perioperative cardiac risk	The cumulative MACE rate was highest in patients with abnormal SPECT and high CACS and lowest in those with normal SPECT findings and low CACS	40 days
Mahla et al. [42]	2001	P	51	69 ± 8	Elective vascular surgery	Assess the predictive value of CACS for perioperative myocardial cell injury	Cardiac troponin T elevations occurred only in patients with CACS > 1000; six patients with perioperative cardiac troponin T elevations had a 2.5-fold higher CACS than those without	7 days

Abbreviations: CACS, coronary artery calcium score; CAD, coronary artery disease; CCTA, coronary computed tomography angiography; ICA, invasive coronary angiography; MACE, major adverse cardiovascular events; MPI, myocardial perfusion imaging; NPV, negative predictive value; OLT, orthotopic liver transplant; R, retrospective; RCRI, Revised Cardiac Risk Index; P, prospective; SPECT, single-photon emission computed tomography.

## Data Availability

No new data were created or analyzed in this study. Data sharing is not applicable to this article.
